# A case of successful treatment with antiretroviral therapy for HIV in a patient with marked liver dysfunction

**DOI:** 10.1016/j.heliyon.2022.e11550

**Published:** 2022-11-14

**Authors:** Hirofumi Fukuda, Yosuke Kondo, Sohji Nishina, Hironobu Ohumi, Yasuyuki Tomiyama, Akihiko Kanki, Hidemitsu Sotozono, Seiya Hashimoto, Harunoshin Yasui, Risa Shimizu, Keisuke Hino, Eisei Kondo, Hideho Wada

**Affiliations:** a Department of Hematology, Kawasaki Medical School, Kurashiki, Japan; b Department of Hepatology and Pancreatology, Kawasaki Medical School, Kurashiki, Japan; c Department of Radiology, Kawasaki Medical School, Kurashiki, Japan

**Keywords:** Antiretroviral therapy, HIV-Associated cholangiopathy

## Abstract

**Background:**

Human immunodeficiency virus (HIV) infection is often complicated with hepatitis virus infection. Antiretroviral therapy (ART) should be initiated with caution for patients with severe virus- or drug-induced acute hepatitis while considering factors that might interfere with the initiation of therapy.

**Case report:**

Herein, we present a case of a 67-year-old woman who presented with symptoms of severe liver injury of unknown cause. Laboratory examinations revealed HIV infection. The HIV viral load was high, and treatment with ART was considered. However, a liver biopsy could not be performed because of hyperbilirubinemia and the risk of severe hepatic damage. After assessing the risk of further liver damage, ART was safely administered despite hyperbilirubinemia. Treatment with ART could successfully reduce the viral load and bilirubin levels.

**Conclusion:**

ART treatment could be safely used for patients with HIV to reduce the viral load and bilirubin levels while avoiding the risk of liver failure.

## 1 Introduction

Human immunodeficiency virus (HIV) infection often overlaps with hepatitis virus infection. In addition, acute hepatitis due to viral or severe drug-induced liver injury may require careful examination at the initiation of antiretroviral therapy (ART), which may adversely affect drug initiation. Here, we report a case of successful ART in a patient with severe liver injury of unknown cause. An otherwise healthy 67-year-old woman presented with severe fatigue, fever, and jaundice. Her HIV viral load was high, and ART was considered. Magnetic resonance imaging revealed a high-intensity region around the intrahepatic portal vein. However, we could not perform a liver biopsy because of hyperbilirubinemia and the risk of severe hepatic damage. Therefore, ART was not initiated. After excluding viral hepatitis and drug-induced hepatitis, ART was safely administered despite hyperbilirubinemia. During ART, the viral load and bilirubin levels simultaneously decreased. The patient was discharged after 140 days. Antiviral therapy can reduce the viral load and help patients avoid liver failure, which is a clinically beneficial result.

## 2 Case report

An otherwise healthy 67-year-old woman presented to a private clinic with common cold-like symptoms, and the clinician diagnosed her with upper bronchitis. She was prescribed garenoxacin mesylate hydrate, dimemorfan phosphate, and L-carbocysteine.

After 10 days from the appearance of the bronchitis symptoms, she was also presented to another hospital with severe fatigue, fever, and jaundice. Her initial findings were as follows: total bilirubin (T-Bil) 8.1 mg/dL, direct bilirubin (D-Bil) 5.8 mg/dL, alanine aminotransferase (ALT) 222 U/L, asparate aminotransferase (AST) 115 U/L, prothrombin time (PT) 104%, activated partial thromboplastin time (APTT) 29.2 s. Viral hepatitis was suspected due to hepatitis-like symptoms, including acute liver injury, fever, and jaundice; however, serological testing was negative for viral hepatitis, hepatitis B virus (HBV), hepatitis C virus (HCV), Epstein-Barr virus (EBV), and cytomegalovirus (CMV). Although the patient was not a drug user or a commercial sex worker, she was tested for human immunodeficiency virus (HIV), another virus that could cause liver damage, and the antibody test was found to be positive. Her HIV viral load was high (119,036 copies/mL), and required treatment. The patient was transferred to our hospital, and then antiretroviral therapy (ART) was initiated.

Initial laboratory values in our hospital and other liver biomarkers were as follows: T-Bil 10.2 mg/dL, ALT 166 U/L, AST 94 UL, INR 1.64, APTT 27.6 s. Since biliary enzymes were extremely high, we considered that biliary strictures should be differentiated. For diagnosis of biliary stricture, ultrasonography (US), computer tomography (CT), magnetic resonance image (MRI) and magnetic resonance cholangio pancreatography (MRCP) were initially considered to be performed as non-invasive examination methods. However, no stenosis was observed in the US or MRCP of the patient. Abdominal US at our hospital revealed a blunted rim of the liver and no dilation of the intrahepatic and extrahepatic bile ducts. The patient was diagnosed with acute hepatitis. T2-weighted (T2W) MRI of the abdomen showed a high-intensity region around the intrahepatic portal vein, indicating abnormal periportal intensity. No laboratory findings confirming bile duct structure that would identify sclerosing cholangitis were obtained. Bile duct stenosis, which could positively identify the sclerosing cholangitis could not be performed. To confirm bile duct stenosis and other histological abnormalities, liver biopsy was essential for accurate diagnosis; however, liver biopsy could not be performed because of hyperbilirubinemia.

While we considered the condition and timing of ART initiation, her liver condition worsened. We considered that the liver damage might have been induced by some drugs prescribed at the previous hospital; thus, we did not start ART. We tried to reduce cholestasis, using ursodeoxycholic acid (UDCA), but her jaundice worsened. Based on the MRI findings, considering the inflammatory changes in the portal region, prednisolone treatment was started; however, the patient did not respond to the treatment.

She showed a rapid progression to anemia and fever; a bone marrow puncture was performed, which indicated hemophagocytic syndrome (HPS). Serum laboratory data also indicated HPS. Each index of appearance of HPS was elevated, including ferritin 9,923 ng/mL, fibrinogen 429 mg/dL, and triglyceride 525 mg/dL. Based on the HIV viral load, we decided to start ART. We diagnosed the HPS as viral-associated HPS (VAHS) due to the exacerbation of viral infection. To avoid ART-related liver toxicity, we selected Emtricitabine/Tenofovir alafenamide fumarate (FTC/TAF) + Raltegravir (RAL) as the ART. Due to elevated hepatobiliary enzymes the ART regimen was changed to RAL+3TC. With this regimen, T-Bil gradually decreased to 9.0 mg/dL.

Her treatment was changed from RAL+ 3TC to TAF/FTC + RAL on the 104^th^ day post hospitalization because of the improvement in hepatobiliary enzymes. The HIV viral load decreased, and the biliary enzymes were within normal limits. She was discharged on the 140^th^ day post hospitalization.

Written informed consent was obtained from the patient for the publication of this report.

## 3 Discussion

**Figure 1:**
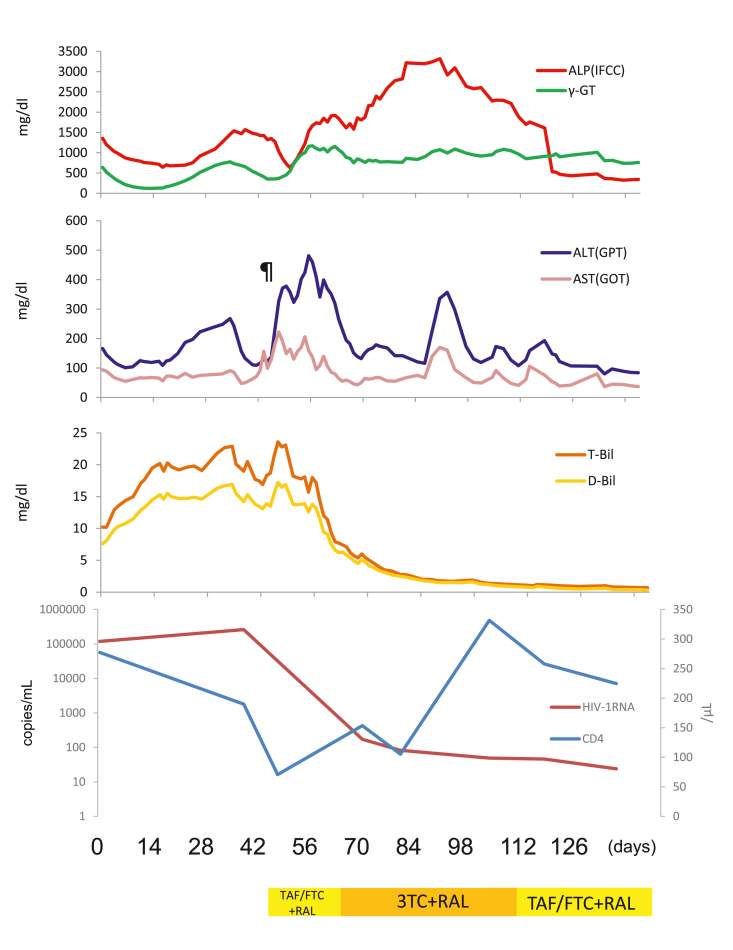
After the initiation of anti-retrovirus therapy (ART), slight hyperbilirubinemia was observed. The ART was successful, and the viral load has tremendously dropped. Further, the viral load and the bilirubin value decreased simultaneously. The increase after a small decline (¶) may be caused by immune reconstruction inflammatory syndrome (IRIS).

This case highlights a candidate treatment for cases with no immediate causes of liver dysfunction in patients with HIV infection. It is advisable to consider various possibilities when considering the biliary enzyme elevations seen in HIV patients. Drug-induced cholangiopathy was excluded because of a report of acetaminophen that was prescribed to treat her (Tajiri et al., 2008). In this case, ART was started after viral hepatitis; thus, drug-induced hepatitis was excluded. In addition to the success of ART, viral load was steadily reduced, and the treatment was safely completed even though the patient developed hyperbilirubinemia. Furthermore, the viral load and bilirubin levels decreased simultaneously (Figure 1). This might support the theory that ARTcan improve the pathological conditions that cause abnormalities in biliary enzymes.

Liver disorders with HIV are often associated with hepatitis virus infection or induced by ART (Joshi et al., 2011). In this case, strong liver disorder and biliary enzyme elevation were seen from an early stage, despite the patient having no relevant medical history. Therefore, this case is very rare.

Cholestasis in patients with HIV infection occurs commonly due to sclerosing cholangitis and AIDS cholangitis. However, no stenosis was observed in the US or MRCP of the patient (Kochar et al., 2010). MRCP in our hospital had a unique finding of periportal abnormal intensity, but this finding is also seen in hepatitis, cholangitis, and primary biliary cirrhosis. Therefore, this finding is not specific to AIDS cholangitis.

AIDS cholangiopathy is a syndrome which involves the obstruction of the bile ducts due to stenosis-related infections. US provides high sensitivity and specificity for the diagnosis of AIDS cholangiopathy (Daly and Padley, 1996). MRCP provides accurate, non-invasive diagnosis of AIDS cholangiopathies through the identification of bile duct stenosis in patients with HIV (Tonolini and Bianco, 2013). Considering that antimicrobial therapy for HIV-associated cholangiopathy is ineffective, ART could be one of the best selection therapies for HIV-associated cholangiopathy. In patients with HIV-associated cholangiopathy, immune reconstitution might be the best choice (De Angelis et al., 2009). Therefore, if the diagnosis is HIV cholangitis, our strategy and treatment are of great significance in this case. Additionally, after starting ART, ALP and GGT levels initially showed a minimal decline, followed by a large increase (Figure 1). This increase might be due to the immune reconstitution inflammatory syndrome (IRIS). Thus, this may provide a medical indication that the patient's immunity is being reconstructed with ART.

A previous report showed a correlation between the number of Kupffer cells and CD4 T cell counts (Balagopal et al., 2009). In this case, liver dysfunction improved with an increase in CD4 lymphocyte count. When considering the symptoms and imaging results of hyperbilirubinemia, normal liver imaging, negative viral, and autoimmune hepatitis studies, this case seemed to be similar to vanishing bile duct syndrome (VBDS). However, few reports have described VBDS in patients with HIV (Oppenheimer et al., 2013). VBDS has a variable clinical course and is associated with cholestasis. VBDS has multiple causes including advanced AIDS, and has been reported as a cause of hyperbilirubinemia associated with HIV. AIDS-related VBDS has a very poor prognosis and often results in liver failure and death. In this case, although the diagnosis of extrahepatic bile duct obstruction was confirmed by imaging studies, a histological diagnosis was not performed. Although a liver biopsy was not performed and the cause of the hyperbilirubinemia has not been determined, if VBDS occurred in this patient, it suggests that ART can be safely administered with the appropriate choice of drugs that do not cause liver damage. This patient had already been confirmed to be HIV positive and was undergoing medical examination for liver dysfunction. If HIV causes liver damage, it is usually due to acute hepatitis caused by hepatitis B, in which case patients will have a high HIV viral load. We also considered the possibility of a combination of primary biliary cholangitis (PBC) and primary sclerosing cholangitis (PSC) due to HIV positivity. However, we were unable to identify whether the virus caused acute hepatitis, autoantibodies, or imaging findings of collagen disease. Therefore, we believe that HIV infected the liver, and the hepatocytes and biliary system were impaired by a very strong immune response. This is supported by the parallel response of the virus, liver enzymes, and bilirubin.

## 4 Conclusion

When administering medical care to HIV-positive patients, there are cases wherein ART might be contraindicated because of drug-related liver disorders, viral liver disorders, or other liver disorders. Although the patient had marked liver dysfunction and no clear cause could be identified, biliary enzymes were rapidly improved after ART initiation. Therefore, it is important to promptly perform antiviral therapy for the treatment of HIV when hepatitis virus infection or drug resistance can be excluded in cases of marked liver dysfunction.

## Declarations

### Author contributions

All authors listed have significantly contributed to the investigation, development and writing of this article.

### Funding statement

This research did not receive any specific grant from funding agencies in the public, commercial, or not-for-profit sectors.

### Data availability statement

No data was used for the research described in the article.

### Declaration of interest's statement

The authors declare no conflict of interest.

### Additional information

No additional information is available for this paper.
